# Cell-based assay for ciliopathy patients to improve accurate diagnosis using ALPACA

**DOI:** 10.1038/s41431-021-00907-9

**Published:** 2021-05-27

**Authors:** Cenna Doornbos, Ronald van Beek, Ernie M. H. F. Bongers, Dorien Lugtenberg, Peter. H. M. Klaren, Lisenka E. L. M. Vissers, Ronald Roepman, Machteld M. Oud

**Affiliations:** 1 Department of Human Genetics, Radboud University Medical Center, Nijmegen, The Netherlands; 2 Radboud Institute for Molecular Life Sciences, Radboud University Medical Center, Nijmegen, The Netherlands; 3 Donders Institute for Brain, Cognition and Behavior, Radboud University Medical Center, Nijmegen, The Netherlands; 4 Department of Animal Ecology and Physiology, Institute for Water and Wetland Research, Radboud University, Nijmegen, The Netherlands

**Keywords:** Translational research, Genetic counselling, Genetics research, High-throughput screening, Diseases

## Abstract

Skeletal ciliopathies are a group of disorders caused by dysfunction of the cilium, a small signaling organelle present on nearly every vertebrate cell. This group of disorders is marked by genetic and clinical heterogeneity, which complicates accurate diagnosis. In this study, we developed a robust, standardized immunofluorescence approach to accurately diagnose a subset of these disorders. Hereto we determined and compared the cilium phenotype of healthy individuals to patients from three different ciliopathy subgroups, using skin-derived fibroblasts. The cilium phenotype assay consists of three parameters; (1) ciliogenesis, based on the presence or absence of cilium markers, (2) cilium length, measured by the combined signal of an axonemal and a cilium membrane marker, and (3) retrograde intraflagellar transport (IFT), quantified by the area of the ciliary tip. Analysis of the cilium phenotypic data yielded comparable and reproducible results and in addition, displayed identifiable clusters for healthy individuals and two ciliopathy subgroups, i.e. ATD and CED. Our results illustrate that standardized analysis of the cilium phenotype can be used to discriminate between ciliopathy subgroups. Therefore, we believe that standardization of functional assays analyzing cilium phenotypic data can provide additional proof for conclusive diagnosis of ciliopathies, which is essential for routine diagnostic care.

## Introduction

Skeletal ciliopathies are a group of rare congenital disorders caused by dysfunction of cilia. As the name implies, all skeletal ciliopathies are characterized by skeletal abnormalities and can additionally present with other organ abnormalities. The majority of skeletal ciliopathies are categorized as short-rib thoracic dysplasia syndromes (SRTD, MIM %208500). The SRTDs include short-rib polydactyly syndrome (SRPS, MIM #263520, #614091, #615503, #269860), Mainzer-Saldino syndrome (MZSDS, MIM #266920), and (Jeune) asphyxiating thoracic dystrophy (ATD, MIM %208500, #611263, #613091, #613819, #614376). In addition, there are three non-SRTD skeletal ciliopathies; Ellis-van Creveld syndrome (EVC, MIM #225500), endocrine-cerebroosteodysplasia (MIM #612651), and cranioectodermal dysplasia syndrome (CED, Sensenbrenner syndrome, MIM #218330, #613610, #614099, #614378). A complicating matter in the accurate diagnosis of patients with a suspected skeletal ciliopathy is the broad phenotypic spectrum, in combination with the overlap in symptoms amongst the skeletal ciliopathy subgroups.

Skeletal features that are observed in almost all patients with a skeletal ciliopathy are a narrow thorax, shortening of the ribs, and abnormal limbs and digits. These features are most severe in SRPS patients, resulting in embryonic or neonatal lethality [1–3]. A close resemblance of this phenotype, but to a milder degree, is seen in patients with ATD and MZSDS. Nonetheless, the majority of deaths in ATD are still attributed to cardiorespiratory failure [4, 5]. Although less common, a narrow rib cage can also be observed in patients with CED and EVC [6]. Other symptoms include pelvic abnormalities, like small ilia and flattened acetabular roofs with ossified spurs. These are seen in patients with ATD and EVC, but again have the most severe presentation in SRPS. Furthermore, the development of the long bones and digits are often affected in patients with a skeletal ciliopathy. For example, polydactyly of the hands and feet are observed in SRPS, ATD, and EVC, but to a lesser extent in CED [4, 5, 7, 8]. On the other hand, CED and EVC are characterized by craniofacial dysmorphologies, which are uncommon in the other skeletal ciliopathy subgroups. The considerable overlap in the skeletal phenotype seen amongst the different subgroups complicates accurate diagnosis, as was shown by the reclassification of SRPS subtypes I and III to ATD subtype 3 [9]. Besides the characteristic skeletal abnormalities, skeletal ciliopathies share numerous non-skeletal features with other ciliopathies. Three important features are renal insufficiency, retinal degeneration, and ectodermal abnormalities. Within the skeletal ciliopathy subgroup renal insufficiency, which can lead to end-stage renal disease is observed in ATD, MZSDS, and CED and retinal degeneration is observed in MZSDS and ATD, while ectodermal abnormalities are only observed in CED and EVC [6]. Because of this broad and overlapping phenotype, diagnosis of patients with a skeletal ciliopathy is often based on the genetic defects underlying the disease.

There is also a significant overlap in genes associated with each of the skeletal ciliopathies, which in turn complicates accurate diagnosis. The majority of skeletal ciliopathy-associated genes are part of the intraflagellar transport A (IFT-A) complex or play a role in the regulation of IFT-A transport. Other disease-associated genes have a role in IFT-B transport or ciliogenesis. The patients with a skeletal ciliopathy presented in this study carry pathogenic variants in genes encoding IFT-A-associated proteins, i.e. *WDR35*, *WDR19*, *IFT43*, and *DYNC2H1*. All of these four genes are known to cause more than one ciliopathy subtype, which can be a skeletal or non-skeletal ciliopathy subtype. Pathogenic variants in *WDR19* are known to underly CED and ATD, but also isolated nephronophthisis and Senior-Løken syndrome [10, 11]. Similarly, genetic defects in *IFT43* can cause CED, SPRS, and isolated retinitis pigmentosa, and *WDR35* is associated with CED and SRPS [8, 12, 13]. Mutations in the *DYNC2H1* gene, encoding a subunit of the IFT-A motor dynein-2, can cause ATD and different subtypes of SRPS [5, 14]. On a molecular level, pathogenic variants in IFT-A complex-associated genes have been reported to reduce cilia abundance and in most cases also showed tip bulging, marked by IFT-B protein accumulation at the tip of the cilia [14–16]. Driven by dynein motor proteins, the IFT-A complex transports proteins in a retrograde manner, from the tip of the cilium down to the base. In contrast, the IFT-B complex facilitates kinesin-dependent anterograde transport, from the ciliary base to the tip. Transport within the cilium is essential for ciliary assembly, disassembly, maintenance, and transport of signaling molecules [17–20]. Consequently, IFT influences the functionality of cilia and thereby plays a pivotal role in embryonic development. IFT performance and other cilium characteristics, such as ciliogenesis and cilium length, could provide additional information about skeletal ciliopathies that may facilitate more accurate diagnosis of patients with a skeletal ciliopathy.

An accurate diagnosis is required to minimize disease burden for patients with skeletal ciliopathies, both to inform the patient of disease progression and to plan proper clinical follow up. Currently, patients with skeletal abnormalities suggestive for a ciliopathy receive whole-exome sequencing or gene panel analysis of the commonly affected ciliopathy genes in order to obtain a genetic diagnosis. In the ~60% of cases where no known pathogenic variant is identified, functional studies are required to confirm the presence of a ciliopathy. These cilium-based studies, however, often have a low reproducibility due to heterogeneity in ciliary parameters, in both controls and patient-derived material. These ciliary parameters include ciliary abundance, length, and IFT88 accumulation at the ciliary tip. The latter is used as a measurement for retrograde transport [14, 21]. The heterogeneity of these parameters is further increased through minimal differences in culturing, cell processing, imaging, and/or analysis methods that are used in reported studies, making functional studies time consuming and difficult to interpret. Therefore, in this study, we set out to develop a method with a standardized cell culture and imaging approach for patient-derived fibroblast with automated analysis of the microscopic images. The advantage of automatization in image analysis is to increase reproducibility, while standardization provides a framework for accurate data interpretation. By using this standardized method, we were able to set analysis standards for healthy cilia parameters including ciliary length, ciliogenesis, and IFT functioning in the cilia. Furthermore, based on these parameters, we were able to reliably distinguish between patient and control cilia and to pinpoint separate clusters of ATD and CED patients.

## Materials and methods

### Editorial policies and ethical considerations

The study was conducted according to the ethical tenets of the Declaration of Helsinki and in agreement with the “Code of Conduct for responsible use of patient-derived material”. For patients whose materials were derived from the Radboudumc Biobank Genetics and Rare Disease (CMO-2018-4985), additional approval was obtained by the local ethics committee of the Radboudumc (CMO-2019-6029). All patient gene variants mentioned in this study are listed in Supplementary Table 1 in accordance with HGVS nomenclature guidelines.

### Fibroblast culture and immunofluorescent staining

Skin-derived fibroblasts were cultured for immunofluorescent analysis of the cilium phenotyping parameters. In brief, upon fixation the cells were incubated with the following primary antibodies: anti-ARL13B, anti-IFT88, anti-acetylated-α-tubulin, and anti-PCNT. Subsequently, the coverslips were microscopically analyzed. A detailed description can be found in the “Supplementary Materials and Methods” section “Fibroblast culture and immunofluorescent staining”.

### Automated cilia analysis using ALPACA

**Fig. 1:**
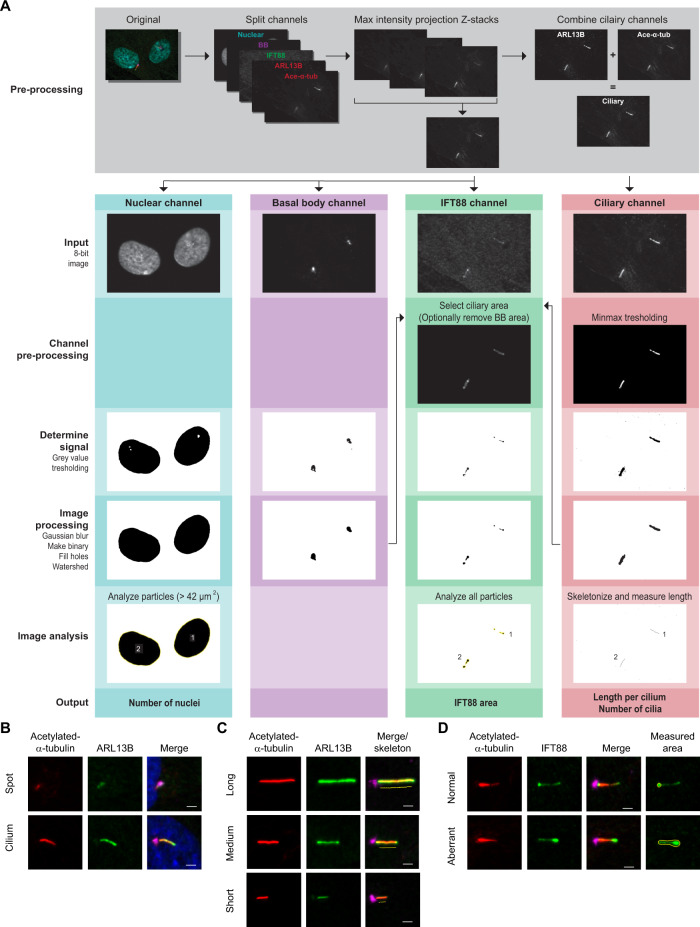
Explanation of ALPACA and cilium phenotype parameters in fibroblasts. **A** Images are pre-proccessed and the split channels are processed and analyzed seperately. To generate the output, the two ciliary channels (ARL13B and acetylated-α-tubulin) are combined to measure cilium length. The number of nuclei and cilia per image are combined to calculate the percentage of ciliated cells. The basal body (BB), ciliary, and IFT channels are used to obtain the area stained for IFT88 within the cilia. **B** Ciliogenesis indicates the percentage of ciliated cells. Cilium axoneme is visualized with acetylated-α-tubulin (red) and ARL13B (green), the base of the cilium is marked with PCNT (pink), and nuclear material with DAPI (blue). **C** The cilium length measurements are based on the combined signal of acetylated-α-tubulin (red) and ARL13B (green). The base of the cilium is marked with PCNT (pink). **D** Retrograde transport is indirectly measured by calculating the surface area of IFT88 (green) at the ciliary tip. The ciliary axoneme is visualized with acetylated-α-tubulin (red) and the base of the cilium is marked with PCNT (pink). Aberrant retrograde transport is illustrated with a cilium showing an increased IFT88 tip surface area compared to a normal cilium tip area. The scale bar indicates 2 µm.

In order to increase reproducibility of the results per fibroblast line and allow screening over a large number of samples, data accumulation was automated using freely available image processing software FIJI (FIJI Is Just ImageJ) [22–24]. The Accumulation and Length Phenotype Automated Cilia Analysis (ALPACA) tool was developed using FIJI in order to automate data generation from a set of microscopic images and optimize recognition of the cellular structures, among which cilia, nuclei, and IFT accumulations (Fig. 1A). The ALPACA tool is available as open source on (https://bitbucket.org/cdoornbos/alpaca). An in-depth description of data analysis and processing by ALPACA can be found in the “Supplementary Materials and Methods”.

### Cilium phenotyping analysis

For each patient and control, we analyzed five microscopic images per parameter; i.e. ciliogenesis, cilium length, and retrograde IFT. The ciliogenesis parameter represents the percentage of cells that have a cilium with an extended axoneme. Ciliogenesis was scored manually per cell, as either ciliated, for which the extended axoneme was visualized by a positive staining for acetylated-α-tubulin + ARL13B + PCNT, or a so-called spot, where a circular staining, positive for acetylated-α-tubulin + ARL13B + PCNT was visible, or no cilium at all (Fig. 1B–D).

The cilium length was calculated using the ALPACA tool, which automatically measures the cilium length based on the ciliary axoneme that is visualized by a combined signal of acetylated-α-tubulin and ARL13B. Last, we studied the performance of retrograde transport in cilia using the retrograde IFT assay parameter of the ALPACA tool. This parameter is based on the surface area of IFT88, after individual assessment of each assay parameter, we combined the data and identified clusters of samples with similar values per parameter.

### Statistical analysis

In all cases the mean and standard deviation (SD) are given and a two-sided *t*-test was used to determine significance, unless stated otherwise. A detailed description of all statistiscal analyses can be found in the “Supplementary Material and Methods”.

## Results

### Clinical and molecular patient data

Upon availability of patient-derived fibroblasts in the “Radboud Biobank Genetics and Rare Diseases” we collected ten patients with a skeletal ciliopathy for this study. Seven out of the ten patients were previously reported in literature [3–5, 8, 10, 21]. An overview of the genotypes and phenotypes is given in Table 1.

**Table 1:** Genetic and clinical findings in ten patients with a skeletal ciliopathy.

Gene	Variant (ClinVar accession)	Variant prediction	Type of variant	Main clinical features	Reference
*WDR35*	NM_001006657.1:c.25-2A > G; NP_001006658.1:p.Ile9fs*7 (SCV000020180.2)	ClinVar: Pathogenic CADD score 34	CH, nonsense	Skeletal: Narrow thorax, pectus excavatum, brachydactyly, syndactyly (feet), polydactyly (hands), short stature, dolichocephaly, craniosynostosis Facial: Telecanthus, narrow palpebral fissure, unilateral ptosis, hypertelorism, strabismus, low-set ears, everted lower lip, micrognathia Kidneys: Renal insufficiency Other: Joint laxity, teeth abnormalities, recurrent lung infections	Gilissen et al. [8] (patient 1)
	NM_001006657.1:c.1877T > C; NP_001006658.1:p.Glu626Gly (SCV000020181.2)	ClinVar: Pathogenic CADD score 35	CH, missense		
*IFT43*	NM_052873.2:c.1A > G; NP_443105.2:p.Met1_Lys21 del (SCV000045384.3)	ClinVar: Pathogenic CADD score 25	Homozygous, deletion	Skeletal: Narrow thorax, brachydactyly, polydactyly (hands and feet), syndactyly (feet), short long bones, short stature, scaphocephaly, craniosynostosis Facial: Frontal bossing, telecanthus, everted lower lip, micrognathia Kidneys: NPHP, ESRD at 3 years old Other: Joint laxity, liver cirrhosis, teeth abnormalities, nail and hair abnormalities, skin laxity, cardiac abnormalities	Arts et al. [21] (patient 2)
*WDR19*	NM_025132.3:c.2129T > C; NP_079408.3:p.Leu710Ser (SCV000044972.5)	ClinVar: Pathogenic CADD score 28	CH, missense	Skeletal: Narrow thorax, brachydactyly, pectus excavatum, hip dysplasia (recovered), spinal abnormalities Kidneys: NPHP-like nephropathy, renal transplant at 14 years old Other: Retinitis pigmentosa, joint hypermobility, teeth abnormalities, skin laxity, thick nails, bone marrow hypoplasia	Bredrup et al. [10]
	NM_025132.3:c.3307C > T; NP_079408.3:p.Arg1103* (SCV000044973.5)	ClinVar: Pathogenic CADD score 38	CH, nonsense		
*WDR35*	NM_001006657.1:c.932C > A; NP_001006658.1:p.Trp311Leu (SCV001548279.1)	ClinVar: Pathogenic CADD score 28	CH, missense	Skeletal: Short stature, brachydactyly, polydactyly (hands), short long bones Kidneys: Bilateral renal cysts, renal transplant Other: Hyperextensibility in fingers, arthogryposis, retinitis pigmentosa, liver cirrhosis, teeth abnormalities, recurrent lung infections, pancreatic cysts, ovarian cysts	Not previously reported
	NM_001006657.1:c.3396-29_3396-18del; r.spl? (SCV001481988.2)	ClinVar: unknown CADD score 8	CH, missense		
*WDR19*	NM_025132.3:c.20T > C; NP_079408.3:p.Leu7Pro (SCV000044974.3)	ClinVar: Pathogenic CADD score 27	Homozygous, missense	Skeletal: Narrow thorax, brachydactyly, rhizomelic abnormalities of long bones, pelvic abnormalities, short stature, inguinal hernia Facial: Wide nasal bridge, thin upper lip Kidneys: Small, proteinuria, renal transplant at 5 years and 12 years old Other: Enlarged liver, yellow teeth, a supernumerary nipple on left side	Bredrup et al. [10]; de Vries et al. [4] (patient 1)
*DYNC2H1*	NM_001080463.1:c.9044A > G; NP_001073932.1:p.Asp3015Gly (SCV000814721.1)	ClinVar: Pathogenic CADD score 33	CH, missense	Skeletal: Narrow thorax, pectus carinatum, pelvic abnormalities, short stature, thoracic scoliosis Kidneys: No abnormalities Other: Respiratory distress 4 days after birth	Schmidts et al. [5] (patient JATD1); de Vries et al. [4] (patient 4)
	NM_001080463.1:c.1306G > T; NP_001073932.1:p.Glu436* (SCV001548280.1)	ClinVar: Pathogenic CADD score 38	CH, nonsense		
*DYNC2H1*	NM_001080463.1:c.7442G > A; NP_001073932.1:p.Arg2481Gln (SCV001548281.1)	ClinVar: Pathogenic CADD score 24	CH, missense	Skeletal: Narrow thorax, (possible) brachydactyly, pelvic abnormalities, short stature Kidneys: No abnormalities	Schmidts et al. [5] (patient JATD3)
	NM_001080463.1:c.9817C > T; NP_001073932.1:p.Gln3273* (SCV001481983.2)	ClinVar: unknown CADD score 59	CH, nonsense		
*DYNC2H1*	NM_001080463.1:c.9044A > G; NP_001073932.1:p.Asp3015Gly (SCV000027072.3)	ClinVar: Pathogenic CADD score 33	CH, missense	Skeletal: Narrow thorax, brachydactyly, syndactyly of feet, pelvic abnormalities, short stature, thoracic scoliosis Kidneys: No abnormalities	Schmidts et al. [5] (patient JATD2); (de Vries et al. [4] (patient 5)
	NM_001080463.1:c.3459-1G > A; r.spl? (SCV001481984.2)	ClinVar: unknown CADD score 34	CH missense		
*DYNC2H1*	NM_001080463.1:c.7594C > T; NP_001073932.1:p.Arg2532Trp (SCV001548283)	ClinVar: Likely pathogenic CADD score 25	CH, missense	Skeletal: Narrow thorax, short long bones, short stature Kidneys: Renal insufficiency Other: Enlarged liver, respiratory insufficiency, speech problems	Not previously reported
	NM_001080463.1:c.11252A > T; NP_001073932.1:p.Glu3751Val (SCV001481985.2)	ClinVar: unkown CADD score 17	CH, missense		
*DYNC2H1*	NM_001080463.1:c.872G > T; NP_001073932.1:p.Cys291Phe (SCV001481986.2)	ClinVar: unknown CADD score 28	CH, missense	Skeletal: Narrow thorax, polydactyly (feet), syndactyly, short limbs Kidneys: Probably one absent kidney Other: Anhydramnios, lobulated tongue, cleft palate, absent nipples, ambiguous genitalia, cardiac abnormalities	Schmidts et al. [3]
	NM_001080463.1:c.536G > A; NP_001073932.1:p.Trp179* (SCV001481987.2)	ClinVar: unknown CADD score 40	CH, nonsense		
	NM_001080463.1:c.10343T > C; NP_001073932.1:p.Leu3448Pro (SCV001548282.1)	ClinVar: Pathogenic CADD score 28	CH, missense		

An overview of the patient lines that were used, including the pathogenic variant and type of variant (Combined Annotation Dependent Depletion (CADD) score version GRCh37-v1.6) [28]. Most patient lines were previously described in the indicated references. The coding positions of the variants refer to: NM_001080463.1 (*DYNC2H1*), NM_052873.2 (*IFT43*), NM_025132.3 (*WDR19*), NM_001006657.1 (*WDR35*). See Supplementary Table 1 for variant accession numbers in accordance with HGVS nomenclature guidelines.

*ATD* (Jeune) Asphyxiating thoracic dysplasia, *CED* cranioectodermal dysplasia, *CEDL* CED-like, *CH* compound heterozygous, *ESRD* end-stage renal disease, *NPHP* nephronophthisis, *SRPS* short-rib polydactyly syndrome.

### ALPACA validation

To validate if the ALPACA tool recognized the correct cellular structures and measured them accordingly, each function of the tool was validated separately. The length measurements by the ALPACA tool were corrected using an artificial dataset (see Supplementary results). The ALPACA measurements were validated against manual measurements and there were no significant differences between the two methods according to the two-sided *t*-test (*p* = 0.3352) (Supplementary Fig. 3A). Furthermore, the methods were comparable according to the mean-difference method previously described by Bland and Altman [25], since all values of the individual cell lines fell within the mean ± 2 SD range (Supplementary Fig. 3C). Last, the averages in length measurements and the differences between patient and controls samples were comparable between methods (Supplementary Fig. 3E).

For the ALPACA IFT88 measurements the best validation method was to compare the results to manually measured data. A drawback of this approach is that the measured IFT88 area is not the same for both methods. The manual measurements were performed as previously described [14, 21] and only the tip area was evaluated, while the ALPACA tool measures all the accumulations along the ciliary axoneme. Therefore, the two manual and ALPACA measurements were compared using two different methods. First, the IFT measurements were normalized against the average of all control samples. Comparing the normalized ALPACA IFT and manual measurements, it could be concluded that there is no significant difference between the two methods (two-sided *t*-test, *p* = 0.1646) (Supplementary Fig. 3B). Second, the manual and ALPACA measurements were compared using the Bland and Altman method as described for the length measurements, since this method accounts for differences between the methods (Supplementary Fig. 3D) [25]. This analysis showed that the two methods were comparable, since all values of the individual cell lines fell within the mean ± 2 SD lines. Last, the IFT measurements showed the same averages in IFT88 accumulation and differences between patient and controls samples for the normalized IFT data (Supplementary Fig. 3F).

In conclusion, the ALPACA tool can accurately analyze the cilia length and retrograde transport through IFT88 accumulations for microscopic images of fibroblasts in a consistent manner and is less time consuming than manual measurements. The nuclei measurements of the ALPACA tool where validated as well; this validation can be found in the Supplementary results.

### Cilium phenotyping in control fibroblasts yields a reproducible “healthy cilium phenotype”

**Fig. 2:**
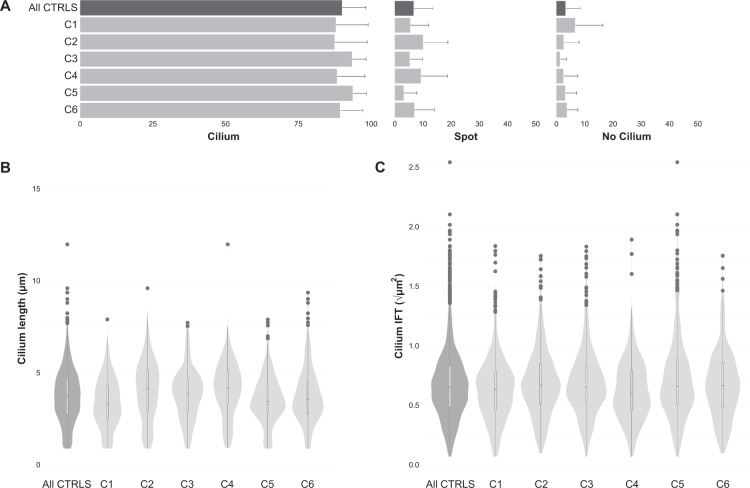
Cilium phenotyping in control cell lines. **A** Ciliogenesis per control, as a percentage of the total number of cells. Cilia are indicated as either present (cilium), spot or not present (no cilium). The top bar of the graph represents the average of all controls, showing 90 ± 8% is ciliated, 7 ± 7% of the cells have a spot and 3 ± 5% is not ciliated. The error bars indicate the SD. **B** Cilium length of all controls in a combined violin-boxplot. The median, Q1, Q3, and range are determined using a Tukey-style boxplot. Outliers (1.5 × IQR) are indicated as dots. The number of observations per length are indicated by the width of the violinplot. The violin-boxplot on the left shows the average of all controls with a median of 3.71 ± 0.04 µm. Individual controls are shown in light gray. **C** The area of the IFT88 accumulations in a combined violin-boxplot as in **B**. The violin-boxplot on the left shows the average of all controls with a median of 0.43 ± 0.01 µm^2^.

Cilium phenotype data were collected from six healthy control individuals. This control dataset was used to create standards for each evaluated parameter (ciliogenesis, cilium length, and retrograde IFT) to determine the “healthy cilium phenotype” (Table 2). Ciliogenesis was calculated based on the percentage of cells that showed an extended axoneme and was measured for each individual cell line. The percentage of ciliated cells was consistently high: always >87% and on average 90%, confirming efficient experimental conditions. In the remaining 10% of cells, we detected either an axoneme that was not extended (“cilium spot”, on average 7% of cells), or cells that had no detectable cilium at all (on average 3% of cells) (Fig. 2). A median cilium length for every cell line was determined using bootstrapping of the ALPACA results given that the data did not show a normal distribution. The “healthy cilium phenotype” showed a cilium length of 3.71 ± 0.04 µm (mean ± SD), based on the calculated medians from six unrelated cell lines. From a different set of images displaying the ciliary localization of IFT88, the IFT parameter was evaluated. The IFT analysis of each control cell line resulted in a healthy cilium phenotype value of 0.43 ± 0.01 µm^2^. The measurements of each individual control cell line are summarized in Table 2. Taken together, the reproducible “healthy cilium phenotype” observed in fibroblasts consisted of 90 ± 8% ciliogenesis, 3.71 ± 0.04 µm in cilium length, and a IFT88 measurement of 0.43 ± 0.01 µm^2^.

**Table 2:** Control lines used for the “healthy cilium phenotype”.

Group	Ciliogenesis	Length	IFT
Controls (*n* = 6)	90 ± 8%	3.71 ± 0.04 µm	0.43 ± 0.01 µm^2^
Control 1	88 ± 11%	3.31 ± 0.10 µm	0.41 ± 0.02 µm^2^
Control 2	87 ± 11%	4.11 ± 0.12 µm	0.45 ± 0.01 µm^2^
Control 3	93 ± 5%	3.84 ± 0.10 µm	0.43 ± 0.03 µm^2^
Control 4	88 ± 10%	4.15 ± 0.14 µm	0.36 ± 0.03 µm^2^
Control 5	94 ± 5%	3.42 ± 0.11 µm	0.44 ± 0.03 µm^2^
Control 6	89 ± 8%	3.54 ± 0.14 µm	0.45 ± 0.02 µm^2^

Cilium phenotype of the individual control lines and their average, represented by the ciliogenesis, cilium length, and the IFT accumulation along the ciliary axoneme.

### Distinctive cilium phenotypes for skeletal ciliopathy subgroups

**Fig. 3:**
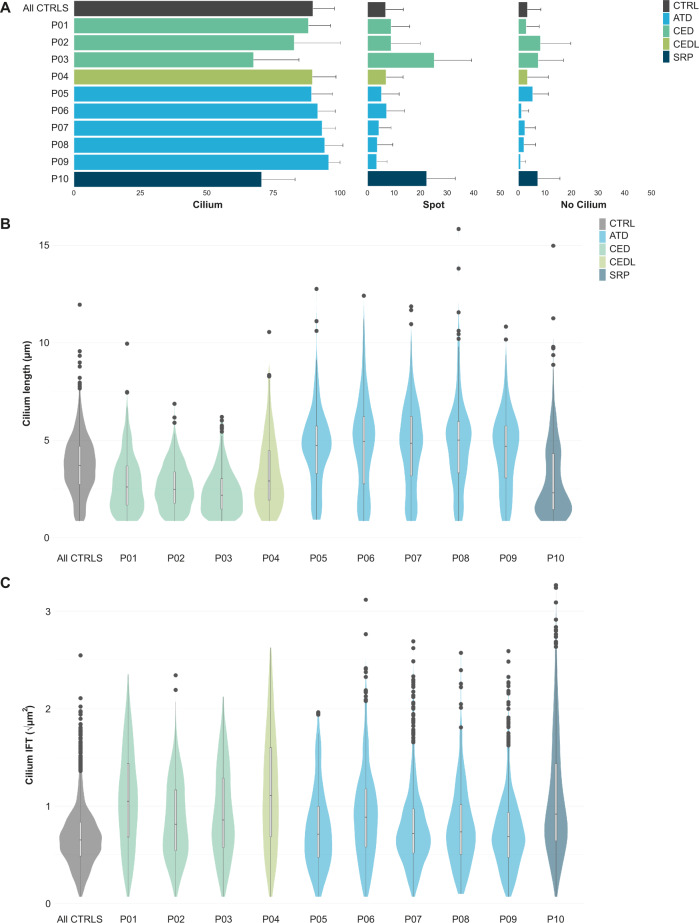
Cilium phenotyping in skeletal ciliopathy patient-derived cell lines. **A** Ciliogenesis per patient, as in Fig. 2A. Patients with SRPS and CED show decreased ciliogenesis with a mean of 71 ± 13% and 80 ± 14%, respectively, in comparison to 90 ± 8% for the controls. Both patient groups show more spot cilia and more unciliated cells. Patients 5–9 with ATD and patient 4 with CEDL showed no major differences in ciliogenesis. **B** Cilium length of all patients, as in Fig. 2B. Patients with ATD showed longer cilia with a median of 4.81 ± 0.06 µm compared to controls (3.71 ± 0.04 µm). Patients with CED showed a shorter cilium with a median of 2.44 ± 0.05 µm, represented by a lower, thicker violin-boxplot. Patients 4 and 10, with CEDL and SRPS, respectively, showed a pear-shaped plot, in which the majority of the cilia are shorter compared to the controls, while a second population of cilia were similar to the controls. Resulting in a median of, respectively, 2.91 ± 0.10 µm and 2.30 ± 0.12 µm. **C** Patient IFT88 measurements, as in Fig. 2C. All patients showed an increased tip area ranging from mild to severe in comparison to the controls. Most of the patients with ATD showed a similar violin-boxplot compared to the controls, but with more outliers at the top, indicating that the majority of cilia have a normal IFT distribution, although some cilia have extremely large IFT accumulations compared to the controls. Patient 10 with SRPS also showed extremely large IFT accumualtions in a subpopulation of the measured cilia, but for this patient the majority of cilia have larger accumulations, resulting in an average tip area of 0.85 ± 0.05 µm^2^ versus 0.43 ± 0.01 µm^2^ for the controls. All three patients with CED showed a longer and smaller violin-boxplot, indicating that the accumulations are larger than in the controls and that there is a broad range in measurements between the cilia. A similar pattern was seen for patient 4 with CEDL.

To determine if potential differences in the collective set of parameters were sufficient to phenotypically distinguish between ciliopathy subtypes, we evaluated this in patients with different skeletal ciliopathy subtypes. All patient-derived fibroblast lines were studied individually, and a cilium phenotype was determined for ten patients (Fig. 3). All five patients with ATD showed normal ciliogenesis (all >89%), an increased cilium length (4.81 ± 0.06 µm), and an increased IFT88 measurement (0.54 ± 0.01 µm^2^) compared to the “healthy cilium phenotype” (Table 3). In the CED cohort consisting of three patients a variable level of ciliogenesis was observed, with patient 3 showing a reduced ciliogenesis of 68 ± 17%, and the other two >83%. All three patients with CED displayed shorter cilia with patient 3 having the shortest cilium length of 2.18 ± 0.09 µm, and patient 1 and patient 2 showed 2.60 ± 0.08 µm and 2.48 ± 0.11 µm, respectively. Similar to the patients with ATD an increased IFT88 measurement was observed in patients with CED, i.e. 0.82 ± 0.03 µm^2^. One patient with SRPS was included in the study who showed reduced ciliogenesis (71 ± 13%), shorter cilia (2.30 ± 0.12 µm), and an increased IFT88 measurement (0.85 ± 0.05 µm^2^).

**Table 3:** Cilium phenotype of patients with a skeletal ciliopathy.

Group/patient	Diagnosis	Gene	Ciliogenesis	Length	IFT
Controls (*n* = 6)			90 ± 8%	3.71 ± 0.039 µm	0.43 ± 0.01 µm^2^
CED (*n* = 3)			80 ± 14%	2.44 ± 0.05 µm	0.82 ± 0.03 µm^2^
Patient 1	CED	*WDR35*	88 ± 8%	2.60 ± 0.08 µm	1.10 ± 0.06 µm^2^
Patient 2	CED	*IFT43*	83 ± 17%	2.48 ± 0.11 µm	0.67 ± 0.03 µm^2^
Patient 3	CED	*WDR19*	68 ± 17%	2.18 ± 0.09 µm	0.74 ± 0.07 µm^2^
Patient 4	CED-like	*WDR35*	90 ± 9%	2.91 ± 0.10 µm	1.24 ± 0.08 µm^2^
ATD (*n* = 5)			93 ± 6%	4.81 ± 0.06 µm	0.54 ± 0.03 µm^2^
Patient 5	ATD	*WDR19*	89 ± 8%	4.73 ± 0.08 µm	0.51 ± 0.05 µm^2^
Patient 6	ATD	*DYNC2H1*	92 ± 7%	4.94 ± 0.13 µm	0.79 ± 0.04 µm^2^
Patient 7	ATD	*DYNC2H1*	93 ± 5%	4.84 ± 0.10 µm	0.42 ± 0.02 µm^2^
Patient 8	ATD	*DYNC2H1*	94 ± 7%	5.01 ± 0.20 µm	0.54 ± 0.03 µm^2^
Patient 9	ATD	*DYNC2H1*	96 ± 4%	4.69 ± 0.11 µm	0.47 ± 0.02 µm^2^
Patient 10	SRPS	*DYNC2H1*	71 ± 13%	2.30 ± 0.12 µm	0.85 ± 0.05 µm^2^

Cilium phenotype of the individual patients, represented by the ciliogenesis, cilium length, and the IFT accumulation along the ciliary axoneme. For the patients with ATD and CED, the median is given per ciliopathy. The average of the controls is given as a reference.

In addition to these three clinically diagnosed patients with CED, we studied a patient with a CED-like (CEDL) phenotype who showed normal ciliogenesis (90 ± 9%), shorter cilia (2.91 ± 0.10 µm), and an increased IFT88 measurement (1.24 ± 0.09 µm^2^).

**Fig. 4:**
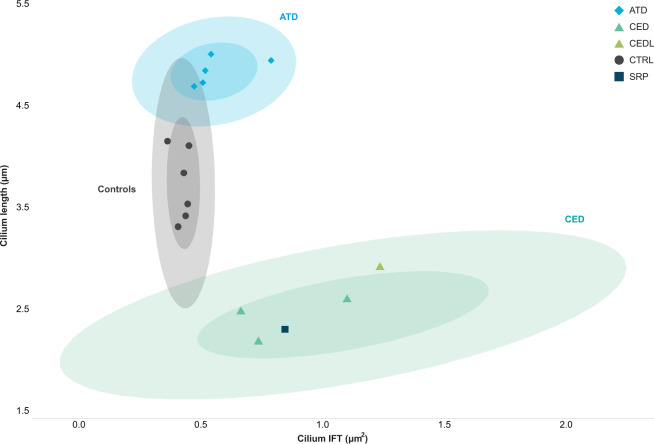
Distinguishable clusters between control and skeletal ciliopathy subgroups. Ciliopathy patient subgroups and controls were clustered based on their cilium length and the IFT88 measurement. The confidence intervals (CI) of 0.5 and 0.9 are indicated per identifiable group, i.e. the control, ATD, and CED cohorts. Dots represent the median per individual. Both the ATD and CED clusters showed slight overlap with the control cluster. Patient 10 with SRPS and patient 4 with CEDL are positioned within the CED cluster.

In summary, compared to the “healthy cilium phenotype”, we observed an increased IFT88 measurement and an abnormal cilium length for all patients in combination with a normal ciliogenesis for the majority of the patients, except for patient 3 with CED and patient 10 with SRPS who showed a reduced number of ciliated cells. A graphical visualization of the data displayed three distinctive clusters, (1) healthy individuals, (2) patients with ATD, and (3) patients with CED (Fig. 4).

## Discussion

The aim of the cilium phenotyping assay was to develop a robust functional assay that could distinguish between healthy controls and ciliopathy patient cohorts, and moreover, classify patients based on their cilium phenotype. We took a systematic approach to overcome the natural variability seen in the assessed cilium phenotype parameters, i.e. ciliogenesis, cilium length, and retrograde IFT. We obtained data from six unrelated control individuals. This yielded comparable and reproducible data resulting in a well-characterized “healthy cilium phenotype”, to which patient phenotypes could be compared. To objectively measure the cilium parameters and thereby improve the robustness and reproducibility of our assay we developed the automated cilium analysis tool ALPACA.

In the present study, we showed that just three cilium phenotype parameters, i.e. ciliogenesis, cilium length, and retrograde IFT, were sufficient to separate skeletal ciliopathy patient data from that of the control cohort. Interestingly, based on these three parameters, the skeletal ciliopathy patient cohort could be further defined into two subgroups, i.e. ATD and CED, that matched the clinical diagnosis of the patients. To our knowledge, this is the first time a systematic and robust assay is presented that enables the classification of patients with a skeletal ciliopathy based on the cilium phenotype of patient-derived fibroblasts.

Using the presented assay, we were able to clearly identify the ciliopathy subtype underlying the phenotype of patient 4 with CEDL. The cilium length and retrograde IFT data of patient 4 are positioned within the CED cluster and are closest to patient 1, who also has pathogenic variants in *WDR35*. This confirmed the already suspected CED diagnosis, giving the patient more clarity about her ciliopathy disorder and diagnostic follow up. On the other hand, the complete cilium phenotype of patient 10 with SRPS showed most resemblance to patient 3 with CED, however clinically patient 10 presented with a more severe and perinatally lethal phenotype. With the current knowledge we are unable to explain this finding.

Publications presenting cilium phenotypic data of patients with a ciliopathy do exist in literature, but often focus on data from only one or two cases with the same syndrome. However, most of the data presented in these publications are in line with our findings. For example, Mill et al., Duran et al., and Merril et al. published that patient-derived cells from patients with SRPS carrying variants in IFT-A associated genes, show aberrant cilium phenotypes consisting of defective retrograde IFT and/or reduced cilium length [13, 14, 26]. The cilium characteristics of patient-derived fibroblasts from patients 1–3 with CED in our study have previously been reported in literature [8, 10, 21]. Cells from patients 1 and 2 showed bulged ciliary tips and for patients 2 and 3 the cilium length was shown to be decreased, which is in line with our findings. Also patients 5–8 with ATD from our study have previously been discussed in publications from Schmidts et al. and Bredrup et al., whom concluded that there were no major differences in cilium length compared to the controls [5, 10]. Strikingly, our results show that the cilium length of all patients with ATD, including patients 5–8, are longer than those observed in controls. This difference could be explained by the fact that in previous studies the inherent variability in cilium length was not taken into account. Due to this variability, comparing patient cell lines to only one or two control cell lines is not sufficient to detect significant differences in length and can lead to misinterpretation of cilium characteristics. This finding supports our standpoint that ample controls and a systematic approach are vital for proper cilium phenotype analysis.

Furthermore, by combining cilium length and IFT88 data, a more detailed analysis could be performed that allowed for the classification of subgroups within the group of skeletal ciliopathy syndromes. Since all ten patients with a skeletal ciliopathy from this study carry pathogenic variants in IFT-A complex-associated genes, it was not surprising to find that they all showed defective retrograde IFT transport. Numerous publications have shown that defects in IFT-A proteins lead to aberrant retrograde IFT, which can be visualized by an accumulation of anterograde IFT proteins at the ciliary tip [21]. In contrast, the clear separation of the ATD and CED subgroups based on the cilium length data was unexpected. Four patients with ATD and one patient with SRPS carry variants in *DYNC2H1* (MIM #613091), based on the location of the variants within the gene and the encoding protein, it is unclear why the clinical symptoms of the patient with SRPS were much more severe than those seen in the patients with ATD. However, it is important to note that unlike any of the patients with ATD who have two inherited variants in *DYNC2H1*, patient 10 with SRPS has three variants, of which NM_001080463.1:c.872G > T, NP_001073932.1:p.Cys291Phe, and NM_001080463.1:c.536G > A, NP_001073932.1:p.Trp179* are located on allele 1, and NM_001080463.1:c.10343T > C, NP_001073932.1:p.Leu3448Pro is located on allele 2. Furthermore, we found that the cilium phenotypes of unrelated patients 5 and 3, diagnosed with ATD and CED, respectively, and who both carry pathogenic variants in *WDR19*, were clearly different. Nonetheless, each patient fitted within its own group of patients with ATD or CED, based on the IFT and length analysis that we performed. Again, we are unable to explain this difference based on the location of the gene variants or the type of variants found. It cannot be excluded that the differences in cilium phenotype seen between patients with variants in the same gene are influenced by other genetic factors that we are currently unaware of. The clear identification of ciliopathy patients based on their cilium phenotype allows us to use this assay to determine whether an undiagnosed patient has a skeletal ciliopathy or not. And in the case of a ciliopathy, determine to which subgroup the patient most likely belongs. The next step will be to study the possible correlations between patient’s clinical characteristics, genotype, and cilium phenotype, however, to do so we need to obtain data from more individuals. In the future these correlations could provide knowledge about organ involvement, disease progression and reveal the link between defective ciliary mechanisms and a specific clinical phenotype. The ALPACA tool was developed to characterize the cilium. The major benefit of an automated tool for cilium length and IFT88 measurements is the considerable reduction in analysis time and the exclusion of human measurements resulting in more robust data. We optimized the cilium length measuremnents by merging two signals prior to the ALPACA analysis. To ensure adequate visualization of the ciliary axoneme a microtubule signal from the acetylated-α-tubulin staining was combined with the ciliary membrane signal from the ARL13B staining. Even so, for two control fibroblast lines the ALPACA tool was unable to correctly measure the length. In these images the displayed cilia still had large gaps in IF staining in the axoneme in combination with a less dense staining of the proximal part of the ciliary axoneme. The cilium phenotyping assay can be used to evaluate new patients by simply performing the assay as described in this study. A technical validation should always be performed by obtaining new measurements of an established control fibroblast line. It is important to note that the exact values for the “healthy cilium phenotype” will have to be determined for each individual laboratory set-up. We recommend researchers to take a similar systematic approach to generate their data and use our results as guidance for data interpretation.

Ciliopathies cover a broad spectrum of syndromes including, but not limited to, skeletal ciliopathies. A limitation of the presented assay, in its current state, is that the three cilium phenotype parameters might not be sufficient to detect other ciliopathy syndromes apart from skeletal ciliopathies. In order to classify other ciliopathies, such as Joubert syndrome (MIM #213300) and Bardet-Biedl syndrome (MIM #209900), the assay needs to be extended with other cilium characteristic parameters, for example SHH signaling or transition zone morphology. To encourage the development of the cilium phenotyping assay by the research community, we made the FIJI tool and its code is freely available (https://bitbucket.org/cdoornbos/alpaca/).

In addition to aiding patient diagnosis, the cilium phenotype assay could play a role in other analyses involving screening of a large number of samples, among which, therapeutic drug studies, ciliogenesis studies or ciliary signaling, or it could be used in combination with other techniques, for example CRISPR genome editing or life cell imaging to study ciliary processes.

The need for more patient-derived functional data, as well as, more tools to analyze a broader scope of ciliopathies is further demonstrated by the increasing amount of data from genetic sequencing techniques. The American College of Medical Genetics and Genomics together with the Association for Molecular Pathology developed standards and guidelines to help with a more uniform interpretation of sequence variants [27]. Amongst other categories, functional studies can provide evidence for the classification of variants, and thus the need for validated, systematic functional assays increases. With the development of tools and assays, as presented in this study, we aim to advance variant interpretation and improve diagnostic care for patients with a ciliopathy.

In conclusion, our results show that systematic analysis of the cilium phenotype can be used to discriminate between control individuals and ciliopathy patients, moreover, specific subgroups of skeletal ciliopathies can be classified based on their cilium phenotype. The presented cilium phenotyping assay is intended to provide guidance for researchers and clinicians working in the ciliopathy field to accurately diagnose patients even in the case of a single patient. Taken together, we developed an assay using patient-derived cells to accurately diagnose patients with a ciliopathy and facilitate genetic counseling allowing the best possible care.

## Supplementary information

Supplemental material
