# Supplementary material for: Cell-based assay for ciliopathy patients to improve accurate diagnosis using ALPACA
Source: Eur J Hum Genet. 2021 May 27;29(11):1677–89. doi: 10.1038/s41431-021-00907-9 (PMC8560805; doi:10.1038/s41431-021-00907-9)
Supplement: Supplementary file 1 — Supplemental material [file 41431_2021_907_MOESM1_ESM.docx]

# Supplementary information

Cell-based assay for ciliopathy patients to improve accurate diagnosis using ALPACA

Cenna Doornbos^1,2^, Ronald van Beek^1,3^, Ernie M.H.F. Bongers^1^, Dorien Lugtenberg^1^, Peter H.M. Klaren^4^, Lisenka E.L.M. Vissers^1,3^, Ronald Roepman^1,2^, Machteld M. Oud^1,3^

# Affiliations

^1^Department of Human Genetics, Radboud University Medical Center, Nijmegen, The Netherlands

^2^Radboud Institute for Molecular Life Sciences, Radboud University Medical Center, Nijmegen, The Netherlands

^3^Donders Institute for Brain, Cognition and Behavior, Radboud University Medical Center, Nijmegen, The Netherlands

^4^Department of Animal Ecology and Physiology, Institute for Water and Wetland Research, Radboud University, Nijmegen, The Netherlands

# Correspondence

Machteld M. Oud, Department of Human Genetics, Radboud University Medical Center, Nijmegen, The Netherlands.
Email: [Machteld.Oud@radboudumc.nl](mailto:Machteld.Oud@radboudumc.nl)

Cenna Doornbos, Department of Human Genetics, Radboud University Medical Center, Nijmegen, The Netherlands.
Email: Cenna.Doornbos@radboudumc.nl

# Supplementary Materials and Methods

## 1.1 Fibroblast culture and immunofluorescent staining

Skin-derived fibroblasts were cultured under standard cell culture conditions in Dulbecco’s modified Eagle’s medium (DMEM) with 20% fetal calf serum (FCS). Cells were cultured on glass coverslips to approximately 90% confluency for immunofluorescent analysis of cilium phenotyping parameters and the experiments were performed in triplicate. Cilia formation was stimulated using serum-starved medium (DMEM with 0.2% FCS) for 48hrs prior to staining. Cells were fixated in 2% paraformaldehyde (PFA) in phosphate buffered saline (PBS) for 20min and permeabilized with 1% Triton-X-100 in PBS for 5min. Subsequently, the cells were blocked for 30min with freshly made 2% bovine serum albumin (BSA) in PBS, followed by a 1hr incubation with the primary antibody at room temperature. Cells were washed with PBS before incubation with the secondary antibody for 1hr at room temperature. The following primary antibodies were used: anti-ARL13B (rabbit polyclonal, Proteintech Group, Manchester, United Kingdom, 1:500), anti-IFT88 (rabbit polyclonal, 13967-1-AP, Proteintech Group, Manchester, United Kingdom, 1:100), anti-acetylated-α-tubulin (mouse monoclonal, T6793, Sigma-Aldrich, Zwijndrecht, Netherlands, 1:1000) and anti-PCNT (PCNT, mouse monoclonal, Abcam, Cambridge, United Kingdom, 1:1000). Secondary antibodies that were used: anti-rabbit Alexa Fluor 488, anti-mouse IgG2B Alexa Fluor 568 and anti-mouse IgG1 Alexa Fluor 647, obtained from ThermoFischer Scientific Waltham, USA. Finally, the coverslips were embedded in Fluoromount-G with DAPI (Southern Biotech, Birmingham, AL, USA) on a microscopic glass slide. Coverslips were microscopically analyzed with a Zeiss Axio Imager Z2 fluorescence microscope (Zeiss, Sliedrecht, Netherlands) equipped with an ApoTome slider using a 63x oil objective. For each coverslip 5 images were taken randomly on the coverslip, consisting of 3-10 z-stacks of 0.24µm to capture every cilium in the imaging area. This systematical imaging approach was used to optimize correct cilia measurements (Supplemental Figure 1). Prior to analysis, the ApoTome-acquired images were converted to deconvolved images using the ApoTome Raw Covert option in ZEN 2012 imaging software (Zeiss, Sliedrecht, Netherlands).

## 1.2 Accumulation and Length Phenotype Automated Cilia Analysis (ALPACA)

The Accumulation and Length Phenotype Automated Cilia Analysis (ALPACA) tool and its code is freely available at ([https://bitbucket.org/cdoornbos/alpaca](https://bitbucket.org/cdoornbos/alpaca/src/master/)). For image analysis, it gathers the data in several steps: image pre-processing, image analysis and generating data output. Each of these steps can be performed sequential by the ALPACA tool or individually by using the ALPACA tool bar for each step.

In order to optimize the recognition of specific cellular structures across a wide variety of different microscopic platforms, the user can alter the signal threshold and signal offset parameters (supplemental material and methods). Next to these two parameters, ALPACA automatically adapts the recognition method based on the meta-data of the image. This means that ALPACA alters the analysis method based on the resolution, and quality of the acquired images. Furthermore, ALPACA generates both numerical and visual output for the user to optimize data interpretation.

The data acquired on cilium length is filtered automatically based on the cut-off value. The cut-off value represents the minimal cilium length that can be measured with the ALPACA tool based on image resolution. Next ALPACA adjusts for the shortening of the cilia during pre-processing.

For image pre-processing each image is split per channel. If multiple channels are named the same, these channels will be merged. In this way, we were able to use a combination of ARL13B and acetylated-α-tubulin to stain the axoneme to minimize gaps in the ALPACA length measurement. Image stacks were merged as maximum intensity projections. All preprocessed images are stored in a separate folder per channel with the original file name.

There are three types of image analysis: count the nuclei per image, measure cilia length or measure IFT accumulations. In case of the combined ALPACA analysis, each channel is analyzed separately for one or several of these analysis methods. To discriminate between relevant signal and background noise, the tool can either automatically threshold the images (by choosing option 0 for automated thresholding), or this can be done manually. Automated thresholding is performed using the Li method (1-3) for the basal body and DAPI channel or the MaxEntropy method (4) for the ciliary and IFT channels.

Afterwards, the images are processed separately per analysis method, however for all three analyses the optimal Gaussian blur (sigma) is automatically determined based on the image resolution, width *sigma* = 0.25/ *pixel width*, with a minimum of sigma = 1. The output data differs per analysis method, but each method includes a visual overlay of the measured objects, as well as, the numerical values per image and the numerical values for the whole set of images.

For counting of the nuclei, the nuclear channel is used and only includes objects larger than 42 μm^2^. ALPACA generates an excel overview of the counted nuclei per image including the area in µm^2^, the circularity of the measured area (0-1, in which 0 represents a straight line and 1 a perfect circle) and the mean of the pixel intensity per measured nucleus. This excel file can be compared to the *.tif* file in which the counted nuclei are numbered in the same manner. Furthermore, the values are summarized per set of images that is analyzed, indicating the total number of nuclei, average area of the nuclei in µm^2^, the mean pixel intensity for the measured areas and average circularity of the nuclei.

For the cilium length measurements, the ciliary channel is used. In the fibroblast measurements, the images of two cilium markers were combined to minimize gaps in the staining. The ALPACA tool can overcome some gaps in the cilium staining and still recognize it as one cilium, however if the gaps are too big or the image quality too poor, the independent structures will be measured as multiple smaller cilia. To optimize the recognition of the cilia, next to the signal threshold described above, the signal offset can be altered to minimize background noise, by increasing the minimal gray value (default range 0-255) prior to using the thresholding algorithms. Nonetheless, in our hands, with optimized staining conditions and sufficient quality of the antibody, this variable can be kept at 0, without affecting cilium length measurements. All length measurements are summarized per image in an excel file and the skeletons per image can be found in the accompanied *.tif* file. The number of cilia that are measured per image divided by the previously determined number of nuclei for this same image will give the percentage of ciliated cells per image.

For measuring the IFT accumulations the channels indicating the IFT signal, basal body and ciliary axoneme are used. In our hands the IFT88 antibody often showed a high background signal, which interfered with measurements. For this reason, the ciliary axoneme channel is used to determine where the cilia are located and to remove the background. In the remaining areas the IFT88 antibody signal intensity was measured. The basal body channel is used to determine cilium orientation. Per image an excel file is generated indicating i.e. the size of each accumulation and circularity, which can be compared to the output *.tif* image, as with the other analysis methods.

## 1.3 Statistical analysis

Ciliogenesis percentages were determined per image, with five images per replicate, three replicates per experiment, and one or two experiments per individual cell line. The mean and standard deviation (SD) were determined for the total number of images per cell line.

Both the length and IFT88 measurements were determined per cilium, with the same number of images as described above for the ciliogenesis parameter. Raw data was tested for normality using the Anderson-Darling test (5) in the *Nortest* package in R version 3.6.2 (6) (Supplemental figure 2). A two-sided T-test was used to determine significance, unless stated otherwise.

For the length measurements, the median values and SD were determined using the bias corrected accelerated bootstrapping method in SPSS (version 25, samples: 1000, confidence intervals: 95%, sampling: simple). Data is shown in a combined Tukey style whisker boxplot and violin plot-density plot per individual cell line. For the IFT88 measurements the median and SD values were calculated using the *Boot* package in R, with 1000 sampling replicates, and ‘ordinary’ simulation. To combine the IFT88 and length measurements, the experiments were grouped per line, and the confidence intervals (CI) of 0.5 and 0.9 were determined as previously described (7).

# Supplementary Results

## 2.1 ALPACA length measurements correction against artificial data and manual measurements

The ALPACA cilium length function was tested in two ways. First, accurate measurement of the tool was assessed using an artificial dataset containing images with bars ranging from 3 pixels to 10 µm in length. Since the width of the cilium skeleton from the skeletonize3D algorithm is 1 to 2 pixels (8, 9), dependent on the curvature, we concluded that the smallest cilium that could be measured by the cilia measurement function is 3 pixels or longer. The longest bar in the artificial data set was set at 10 µm to limit the size of the artificial data set, since the cilia in our screen rarely extend further when measured manually. To account for the differences in cilium width observed in IF images based on the axonemal marker that was used the bars in the artificial test set ranged from the minimal 3 pixels to 1 µm. All images were generated with FIJI with the *‘Generate test files’* script that can be found in the ALPACA package. The measured length from the cilia measurement function was plotted against the theoretical length of each bar and tested for a linear regression model. This indicated that the length measured by the script is directly correlated to the theoretical length according to the linear regression model *y = 0.9648 * x + (-0.5729)* with an *R^2^ = 0.9857* (Supplemental Figure 4A). This model was integrated into the cilia measurement tool to correct the length measured by the cilia measurement script as output for the measurement summary. Last, a cut-off equal to ≥3 pixels for the image specific resolution was applied to all measurements. The corrected length measurements were compared to manual measurements of the same cilia, as previously indicated, and showed no significant differences (Supplemental Figure3). Of note, this correction step also indicated that circular structures could not be measured by the ‘analyze skeleton algorithm’ (8, 9) that is integrated in the cilia measurement function. Each structure with a circularity of 0.8 or higher was measured at a length of 1 or 2 pixels, which is below the cut-off value (Supplemental Figure 4A). Due to this, the ciliogenesis counts were corrected manually, since the cilia that appear as a ‘spot’ will not be recognized by the script.

## 2.2 Validating nuclear ALPACA measurements

The ALPACA results of the ‘nuclei count’ were validated against manual measurements performed by two independent researchers. The number of cells were counted over 20 randomly picked images of control or patient samples and counted double blind. With a two-sided, paired T-test it could be determined that there was no significant difference between the number of nuclei that were counted by the ALPACA tool compared to the average of the manual measurements (Supplemental Figure 4B). Furthermore, it was of interest to see that when the average of the manual measurements was indicated as the true number of nuclei, the difference of the ALPACA measurements with this mean was smaller than those of the individual researchers compared to the mean. The standard error of the mean (SEM) for the ALPACA measurements was approximately half of the manual measurements. Therefore, it could be concluded that the ALPACA tool not only correctly measures the number of cells per image, but it does this more consistently than measurements between individual researchers.

# Supplementary Tables

### **Supplemental Table 1. Patient gene variants**

All previously reported patient variants, including Accession Number in standard HGVS nomenclature.

| **HGVS_transcript** | **HGVS_RefSeqGene** | **HGVS_Predicted_Protein** | **HGVS_Genomic_GRCh37** | **Gene_Symbol** | **HGNC_Gene_ID** |
| --- | --- | --- | --- | --- | --- |
| NM_025132.3:c.20T>C | NG_031813.1:g.8336T>C | NP_079408.3:p.(Leu7Pro) | NC_000004.11:g.39187359T>C | WDR19 | HGNC:18340 |
| NM_001080463.1:c.9044A>G | NG_016423.1:g.116290A>G | NP_001073932.1:p.(Asp3015Gly) | NC_000011.9:g.103091449A>G | DYNC2H1 | HGNC:2962 |
| NM_001080463.1:c.1306G>T | NG_016423.1:g.16552G>T | NP_001073932.1:p.(Glu436Ter) | NC_000011.9:g.102991711G>T | DYNC2H1 | HGNC:2962 |
| NM_001080463.1:c.7442G>A | NG_016423.1:g.87091G>A | NP_001073932.1:p.(Arg2481Gln) | NC_000011.9:g.103062250G>A | DYNC2H1 | HGNC:2962 |
| NM_001080463.1:c.9817C>T | NG_016423.1:g.132107C>T | NP_001073932.1:p.(Gln3273Ter) | NC_000011.9:g.103107266C>T | DYNC2H1 | HGNC:2962 |
| NM_001080463.1:c.3459-1G>A | NG_016423.1:g.50264G>A | NP_001073932.1:p.? | NC_000011.9:g.103025423G>A | DYNC2H1 | HGNC:2962 |
| NM_001080463.1:c.7594C>T | NG_016423.1:g.87720C>T | NP_001073932.1:p.(Arg2532Trp) | NC_000011.9:g.103062879C>T | DYNC2H1 | HGNC:2962 |
| NM_001080463.1:c.11252A>T | NG_016423.1:g.198798A>T | NP_001073932.1:p.(Glu3751Val) | NC_000011.9:g.103173957A>T | DYNC2H1 | HGNC:2962 |
| NM_001006657.1:c.25-2A>G | NG_021212.1:g.5840A>G | NP_001006658.1:p.? | NC_000002.11:g.20189045T>C | WDR35 | HGNC:29250 |
| NM_001006657.1:c.1877A>G | NG_021212.1:g.49337A>G | NP_001006658.1:p.(Glu626Gly) | NC_000002.11:g.20145548T>C | WDR35 | HGNC:29250 |
| NM_052873.2:c.1A>G | NG_031957.1:g.5035A>G | NP_443105.2:p.(Met1?) | NC_000014.8:g.76452130A>G | IFT43 | HGNC:29669 |
| NM_025132.3:c.2129T>C | NG_031813.1:g.54540T>C | NP_079408.3:p.(Leu710Ser) | NC_000004.11:g.39233563T>C | WDR19 | HGNC:18340 |
| NM_025132.3:c.3307C>T | NG_031813.1:g.90637C>T | NP_079408.3:p.(Arg1103Ter) | NC_000004.11:g.39269660C>T | WDR19 | HGNC:18340 |
| NM_001006657.1:c.932G>T | NG_021212.1:g.25568G>T | NP_001006658.1:p.(Trp311Leu) | NC_000002.11:g.20169317C>A | WDR35 | HGNC:29250 |
| NM_001006657.1:c.3396-29_3396-18del | NG_021212.1:g.81387_81398del | NP_001006658.1:p.? | NC_000002.11:g.20113489_20113500del | WDR35 | HGNC:29250 |
| NM_001080463.1:c.872G>T | NG_016423.1:g.13306G>T | NP_001073932.1:p.(Cys291Phe) | NC_000011.9:g.102988465G>T | DYNC2H1 | HGNC:2962 |
| NM_001080463.1:c.536G>A | NG_016423.1:g.10780G>A | NP_001073932.1:p.(Trp179Ter) | NC_000011.9:g.102985939G>A | DYNC2H1 | HGNC:2962 |
| NM_001080463.1:c.10343T>C | NG_016423.1:g.151100T>C | NP_001073932.1:p.(Leu3448Pro) | NC_000011.9:g.103126259T>C | DYNC2H1 | HGNC:2962 |

# Supplementary Figures

### Supplemental Figure 1. Points of attention for imaging and image analysis


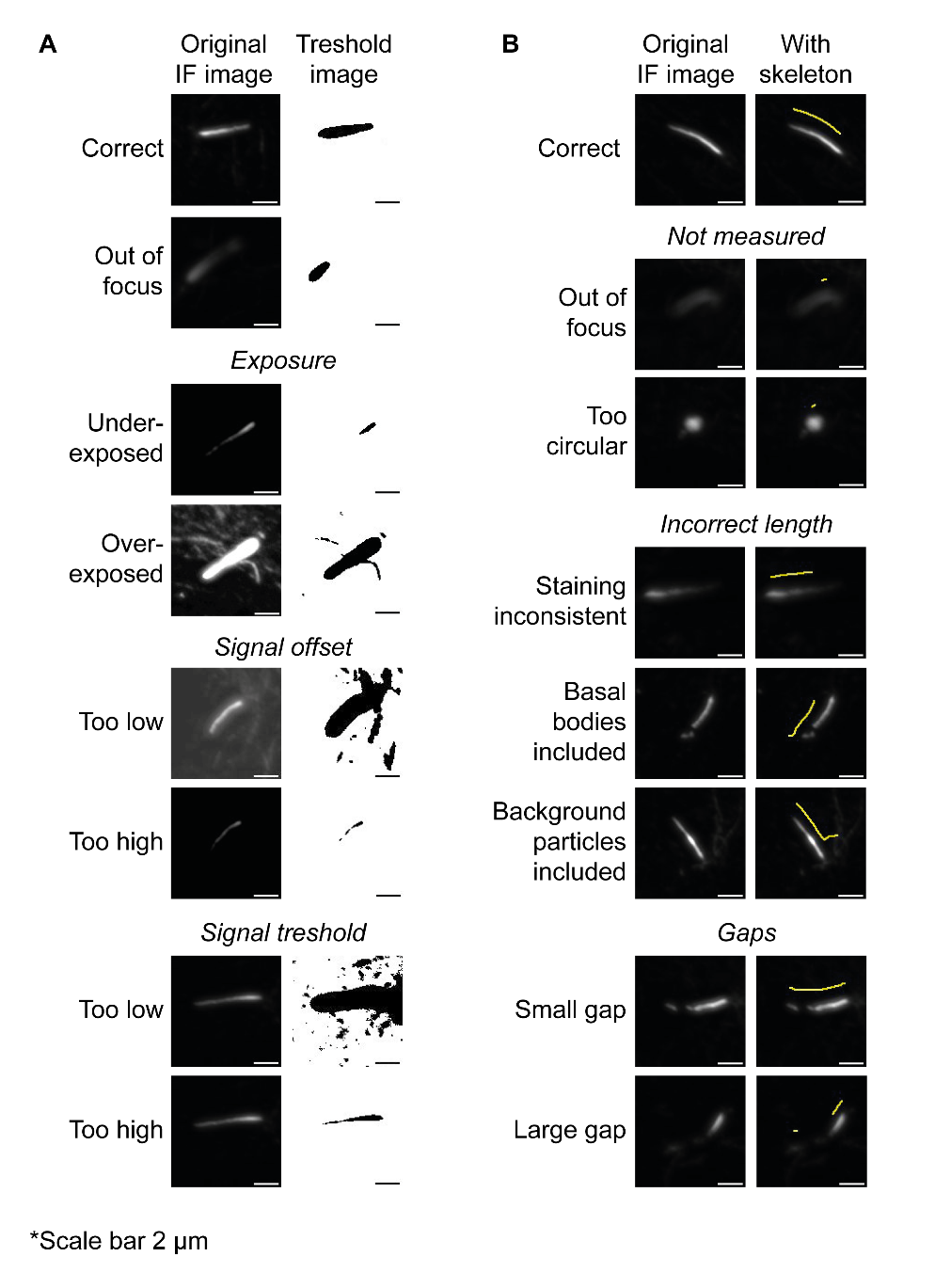
**A**: Examples for correct cilia imaging and analysis. The original image is shown on the left and its thresholded image on the right. The upper rows indicate points of attention during microscopic imaging. Take image stacks to make sure all cilia are within focus and set the exposure correctly to prevent the cilia from being measured too long or too short. The lower rows indicate the importance for correct use of signal offset and signal threshold for the automated cilia length measurements. **B**: attention points to access the quality of automated image analysis. The original image is shown on the left and the same cilium with its skeleton in yellow is shown on the right. The skeleton has an offset for clarification. Cilia that are out of focus or too circular will be measured as a small dot and therefore will be discarded during analysis. In case the staining is inconsistent throughout the cilium they will be measured too short. When the basal bodies are stained by the axonemal marker or if the signal offset and signal threshold settings are incorrect, the measured cilia length might be too long. If the cilia contain gaps that are too wide, these will not be recognized as one cilium, but rather measured as two smaller cilia. Small gaps will be covered correctly by the automated cilium length measurement. Scale bar = 2 μm.

###
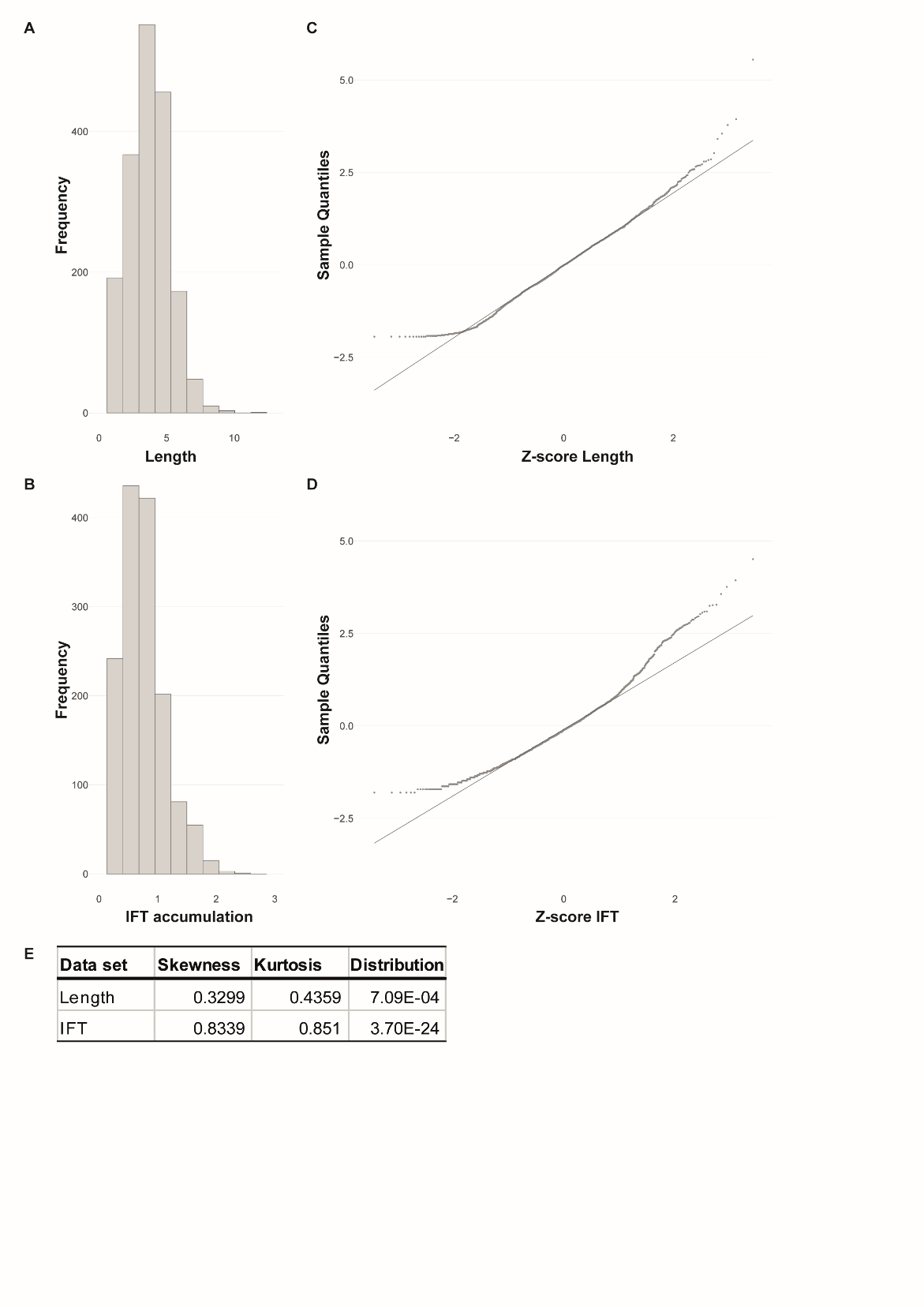
Supplemental Figure 2. Data distribution

Distribution of the length and IFT88 measurements. **A**,**B**: Histogram of the length (A) and IFT88 (B) measurements. In both cases the data showed an asymmetrical distribution with a longer tail towards the right. **C**,**D**: QQ-plot of the length (C) and IFT88 (D) measurements. The line indicates the hypothetical distribution of the data in case of a normal distribution. For both datasets the data distribution does not follow a normal distribution, since the tails on the left and right are above the line. **E**: Numerical values for the data distribution. The positive skewness indicates a more right-tailed data distribution, as seen in the histograms, where the IFT88 set is less symmetrical than the length set, since it diverges further from 0. The positive kurtosis of both datasets indicates a more heavy-tailed distribution compared to a normal distribution, for which again the IFT88 set is less normally distributed than the length dataset, since the kurtosis diverges further from 0. The distribution was determine using the Anderson-Darling test and confirms that both datasets are not normally distributed since p < 0.05.

### Supplemental Figure 3. ALPACA validation


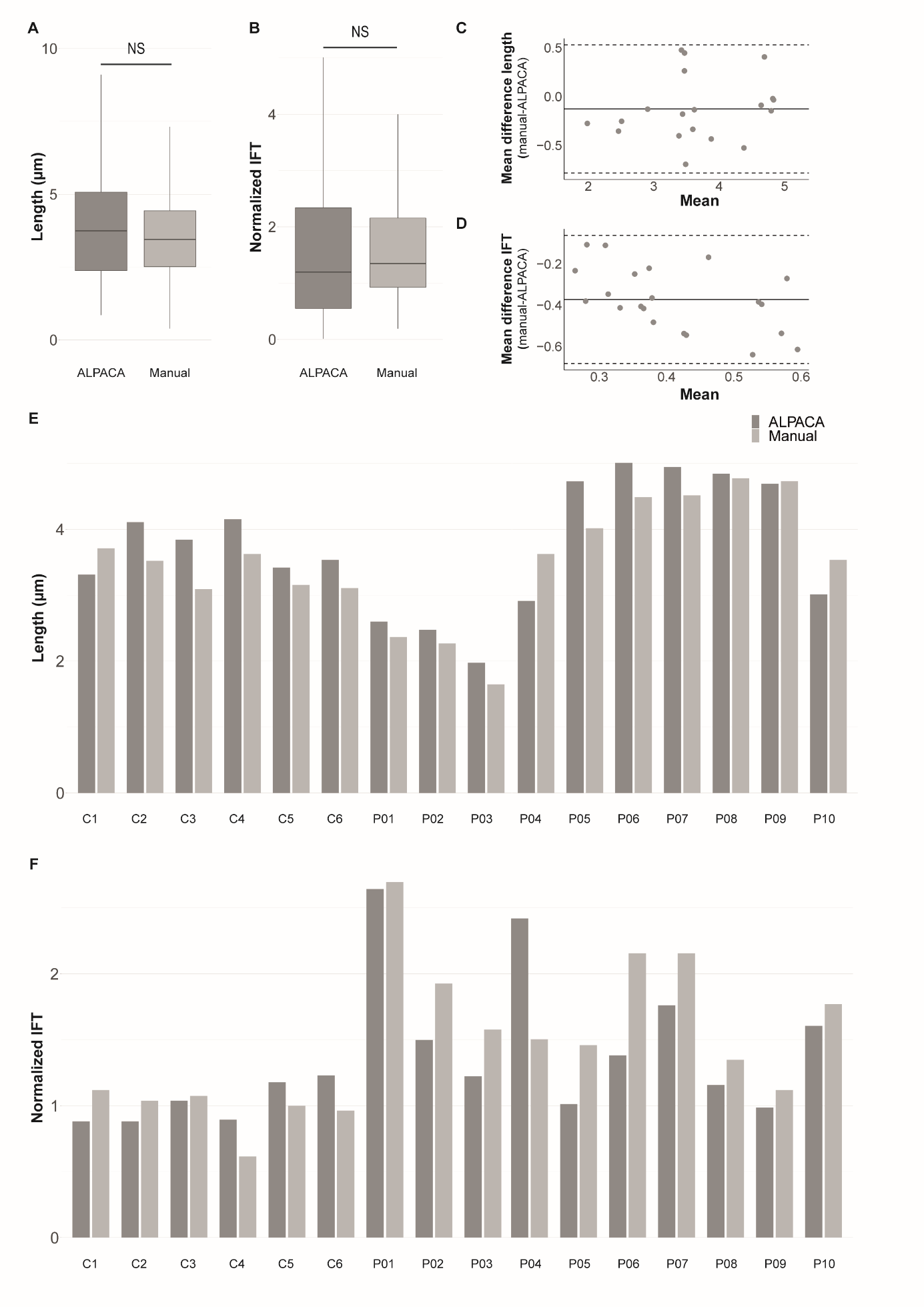


The length and IFT88 measurements of the ALPACA tool were validated against manual measurements. **A**,**B**: Boxplots of the ALPACA measurements compared to the manual measurements for both the length and IFT datasets. For the IFT88 measurements the data was normalized against the average of all controls per method. There is no significant difference between the two methods for both datasets as determined by a paired T-test (p = 0.3352 and p = 0.1646 respectively). **C**,**D**: Bland & Altman plot of the mean difference between the manual and ALPACA measurements for each of the cell lines. The solid line indicates the ‘mean’ of all lines, the upper dashed line represents the ‘mean + 2SD’ and the lower dashed line the ‘mean – 2SD’. All cell lines remain within the mean ± 2SD lines indicating that the manual and ALPACA measurement methods give comparable results for both length and IFT measurements. **E**,**F**: Distribution of the data for the ALPACA compared to the manual measurements for both the length and IFT data. In both cases the two methods show similar measurements in length or IFT88 measurements, independent of the method that was used.

### Supplemental Figure 4. Supplemental results

**
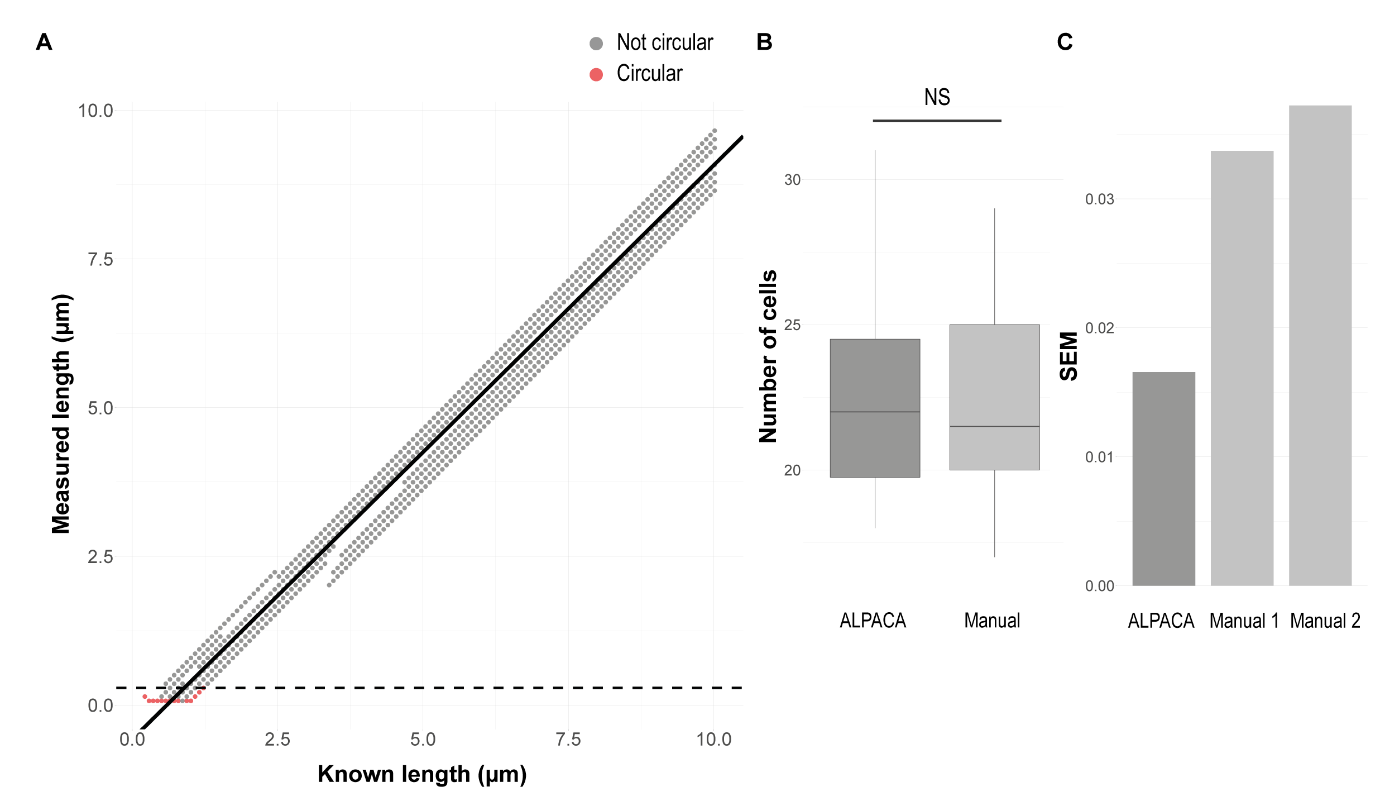
**

**A**: The individual measurements of the artificial dataset with known length (µm) of the drawn cilia bar is compared to the length measured (µm) by the ALPACA length measurement function. Objects with a circularity >0.80 are indicated in red and all other bars are indicated in grey. All data points align to a linear model of y = 0.9648 * x + (-0.5729) with an R2 of 0.9857, indicated by the black line. The black dotted bar indicates that the minimal length that can be measured is >2 pixels, which is equal to 0.144µm for the images resolution that was used. **B**,**C**: Validation of the ALPACA nuclei measurements. There was no significant difference between the ALPACA automated measurements and the combined manual measurements as determined by a two-sided, paired T-test (p = 0.07) (**B**). The standard error of the mean (SEM) for the ALPACA and manual measurements, in which the mean is determined as the mean of the manual measurements. The manual measurements of the two independent researchers (manual 1 and manual 2) show a SEM that is twice as high compared to the ALPACA tool, indicating that the error in measurement made by the tool is smaller than the error between two individual manual measurements (**C**).

# References

1. Li CH, Lee CK. Minimum Cross Entropy Thresholding. Pattern Recognition. 1993;26(4):617-25.

2. Li CH, Tam PKS. An Iterative Algorithm for Minimum Cross Entropy Thresholding. Pattern Recognition Letters. 1998;18(8):771-6.

3. Sezgin M, Sankur B. Survey over Image Thresholding Techniques and Quantitative Performance Evaluation. Journal of Electronic Imaging. 2004;13(1):146-65.

4. Kapur JN, Sahoo PK, Wong ACK. A New Method for Gray-Level Picture Thresholding Using the Entropy of the Histogram. Graphical Models and Image Processing. 1985;29(3):273-85.

5. Thode HC. Normality Tests. In: Lovric M, editor. International Encyclopedia of Statistical Science. Berlin, Heidelberg: Springer Berlin Heidelberg; 2011. p. 999-1000.

6. R Core Team. R: A language and environment for statistical computing. 3.6.2 ed. Vienna, Austria: R Foundation for Statistical Computing; 2019.

7. Fox J, Weisberg S. An R Companion to Applied Regression. 3rd ed: SAGE Publications, Inc; 2018 September 2018.

8. Arganda-Carreras I, Fernandez-Gonzalez R, Munoz-Barrutia A, Ortiz-De-Solorzano C. 3D reconstruction of histological sections: Application to mammary gland tissue. Microsc Res Tech. 2010;73(11):1019-29.

9. Polder G, Hovens HLE, Zweers AJ. Measuring shoot length of submerged aquatic plants using graph analysis. In: Proceedings of the ImageJ User and Developer Conference 2010, Mondorf-les-Bains, Luxembourg, 27-29 October 2010 - Luxembourg : Centre de Recherche Public Henri Tudor. 2010:p. 172 - 7.
